# Effect of a digital patient motivation and support tool on CPAP/APAP adherence and daytime sleepiness: a randomized controlled trial

**DOI:** 10.1007/s41105-023-00479-9

**Published:** 2023-08-17

**Authors:** Christian Franke, Franziska Piezonna, Regina Schäfer, Alexander Grimm, Lisa-Marie Loris, Matthias Schwaibold

**Affiliations:** 1 Medizinisches Versorgungszentrum GbR Sonneberg, 96515 Sonneberg, Germany; 2 Loewenstein Medical Technology GmbH+Co. KG, 76135 Karlsruhe, Germany

**Keywords:** Adherence, Continuous positive airway pressure, Obstructive sleep apnea, Digital patient support, Prisma APP, Telehealth

## Abstract

The purpose of this study was to assess the effect of a digital patient support (DPS) tool, complementary to standard care on continuous or automatic positive airway pressure (auto)CPAP adherence and daytime sleepiness after 12 weeks in patients diagnosed with severe obstructive sleep apnea (OSA). All patients with apnea–hypopnea index (AHI) ≥ 30 per hour were prospectively included and randomized to receive standard care (SC) or standard care with personalized DPS via a mobile app prototype version (SC + DPS). Patients in the SC + DPS arm received additionally automated feedback on their therapy, motivational messages and therapy recommendations. 100 patients completed the study (SC: 50, SC + DPS: 50). No differences were found in characteristics of SC vs. SC + DPS (mean ± SD) for age (53.9 ± 10.8 vs. 51.7 ± 12.3 years), initial diagnostic apnea–hypopnea index (51.1 ± 15.5 vs. 50.9 ± 17.7 events/h), BMI (33.8 ± 6.7 vs. 33.5 ± 4.5 kg/m), and Epworth Sleepiness Scale (ESS) baseline score (9.5 ± 4.2 vs. 9.1 ± 5.2). After 12 weeks, mean ESS score was significantly lower (SC: 7.6 ± 4.1 vs. SC + DPS: 5.5 ± 3.9; *p* = 0.006) in the SC + DPS group vs. standard care group. Therapy adherence was significantly higher (SC: 268.7 ± 122.1 vs. SC + DPS: 338.8 ± 106.8 min; *p* = 0.002) in the SC + DPS group compared to standard care group. No difference was found in the residual AHI between both groups. However, SC + DPS group showed a trend towards fewer phases with increased leakage compared to SC group. Intention-to-treat analysis (112 (56/56) patients) showed similar results. After 12 weeks, (auto)CPAP adherence and daytime sleepiness improved significantly in patients with severe OSA using the digital patient support tool.

**Clinical Trial Registration (retrospectively registered):** Registry: NCT05440279; Title: Effects of Telemedical Support on Therapeutic Results of CPAP Patients; URL: https://clinicaltrials.gov/ct2/show/NCT05440279; Date of registration: June 30, 2022.

**Supplementary Information:**

The online version contains supplementary material available at 10.1007/s41105-023-00479-9.

## Introduction

Obstructive sleep apnea (OSA) is a sleep-related breathing disorder with negative consequences on health and highly prevalent in adults [1, 2].

Positive airway pressure (PAP) therapy is the gold standard in treating OSA. Mostly used therapy modes are: continuous PAP (CPAP) and automatic PAP (APAP) mode.

Studies have shown that increased PAP adherence results in decreased daytime sleepiness, enhanced functional outcomes and memory improvement [3–5], respectively, a greater reduction in blood pressure [6]. Therefore, optimizing PAP therapy adherence is crucial for therapy success.

Many PAP users quit during initial therapy phase. Up to 50% discontinue by 12 months [7, 8].

Therapy adherence is affected by patient demographics and physiology [9–12] as well as patients’ willingness or ability to change lifestyle and mindset, experience of side effects (e. g. mask discomfort, sleep disruption) or psychological factors [13–15].

An important predictive factor of low adherence is bad experience during the initial weeks of therapy [16, 17].

Patient-centered psychological approaches have a significant impact on adherence: perceived self-efficacy relates to PAP adherence [18] and patient´s understanding of the underlying disorder influences adherence and outcomes [19].

PAP device data on adherence, mask leaks, residual apneas are very accurate [20] and can be used to offer digital self-monitoring options for patients, which have been shown to be appreciated [21].

This indicates that digital support has the potential to positively affect PAP adherence.

Literature reviews show an increase of average use in communication-technology-based therapy compared to usual care [22–24]. As evidence for effects is low and telemedicine technologies are becoming more apparent [25], randomized controlled trials are needed.

## Methods

Following the design of a monocentric, prospective, randomized controlled trial, two methods of 12 week follow-up therapy after (auto)CPAP treatment initiation have been compared: standard care (SC) and standard care with personalized digital patient support (DPS) via a prototype version of a later mobile application (“prisma APP”) (SC + DPS). The primary study endpoint was the improvement of self-reported sleepiness (Epworth Sleepiness Scale, ESS [26]) in both groups after 12 weeks of (auto)CPAP therapy.

Secondary endpoints included (1) mean nightly (auto)CPAP adherence and proportion of days with average usage > 4 h, (2) Functional Outcomes of Sleep Questionnaire (FOSQ) score [27, 28], (3) Apnea–Hypopnea-Index (AHI), (4) leakage, (5) periods of stable respiration (defined as even breathing without apneas nor hypopneas and even without sub-hypopnea variations as potential indicator for unfragmented NREM sleep, the parameter showing moderate correlation to N3 sleep in unpublished pilot data), (6) patient satisfaction with treatment, (7) number of phone contacts and on-site appointments and (8) drop-out rate. Stable respiration serves as an indicator of physically restorative deep sleep when muscles relax, blood pressure and breathing rate decline.

### Subjects

Patients who were referred to receive (auto)CPAP devices (prisma SMART/prisma SOFT, Löwenstein Medical Technology GmbH + Co. KG, Hamburg, Germany) for OSA treatment were admitted as potential recruits to a German Sleep Lab (Sonneberg) between February 2019 and October 2021.

Inclusion criteria were age 18–80 years, PAP-naïve, confirmed severe OSA (AHI > 30/h) diagnosis based on polysomnography (PSG) (MiniScreen PRO with Software MiniscreenViewer, Dr. Fenyves & Gut, Rangendingen, Germany and according to AASM scoring manual version 2.4), as well as written informed consent to participate in the study, including a data protection statement.

Exclusion criteria were the presence of a contraindication to PAP therapy, participation in another trial influencing automated electronic support, lack of possibility to receive emails or use electronic means of communication, or lack of patient consent.

### Sample size calculation and randomization

Literature indicates that patients with untreated OSA often range between 12 to 14 points on the Epworth Sleepiness Scale (ESS) on average [29]. The ESS score is a medical parameter to measure the severity of daytime sleepiness. It is categorized in five sections defined as lower normal daytime sleepiness (0–5 points), higher normal daytime sleepiness (6–10), mild excessive daytime sleepiness (11–12), moderate excessive daytime sleepiness (13–15) and severe excessive daytime sleepiness (16–24). CPAP therapy can reduce the ESS score depending on adherence and optimized therapy pressure setting down to 6 points on average [26, 30, 31].

With an estimated standard deviation of 1.5 ESS points in each arm we defined an additional reduction of 1 ESS point in the intervention arm based on the complementing digital support as clinically relevant [29]. The null hypothesis was that the changes in ESS Score are not significantly different between the two groups. In an a priori power analysis, we calculated an effect size *d* = 0.666667 based on the estimated standard deviation and the extra reduction of ESS in the intervention group. Using a one-sided test, an alpha significance level of 0.005, a power of 0.95 and an allocation ratio of 1:1 the power analysis resulted in a sample size of 50 each arm to disprove the null hypothesis. We estimated a drop-out rate of 30% and defined an overall sample size of 130 (65 per arm).

A drop-out was defined as presence of one of the following factors: the primary endpoint data was not available, e. g. when a patient did not attend the follow-up visit to complete the ESS score; there were final discrepancies between the collected data and the source data; there was an exchange of the responsible sleep center or therapy type; informed consent was revoked.

**Fig. 1:**
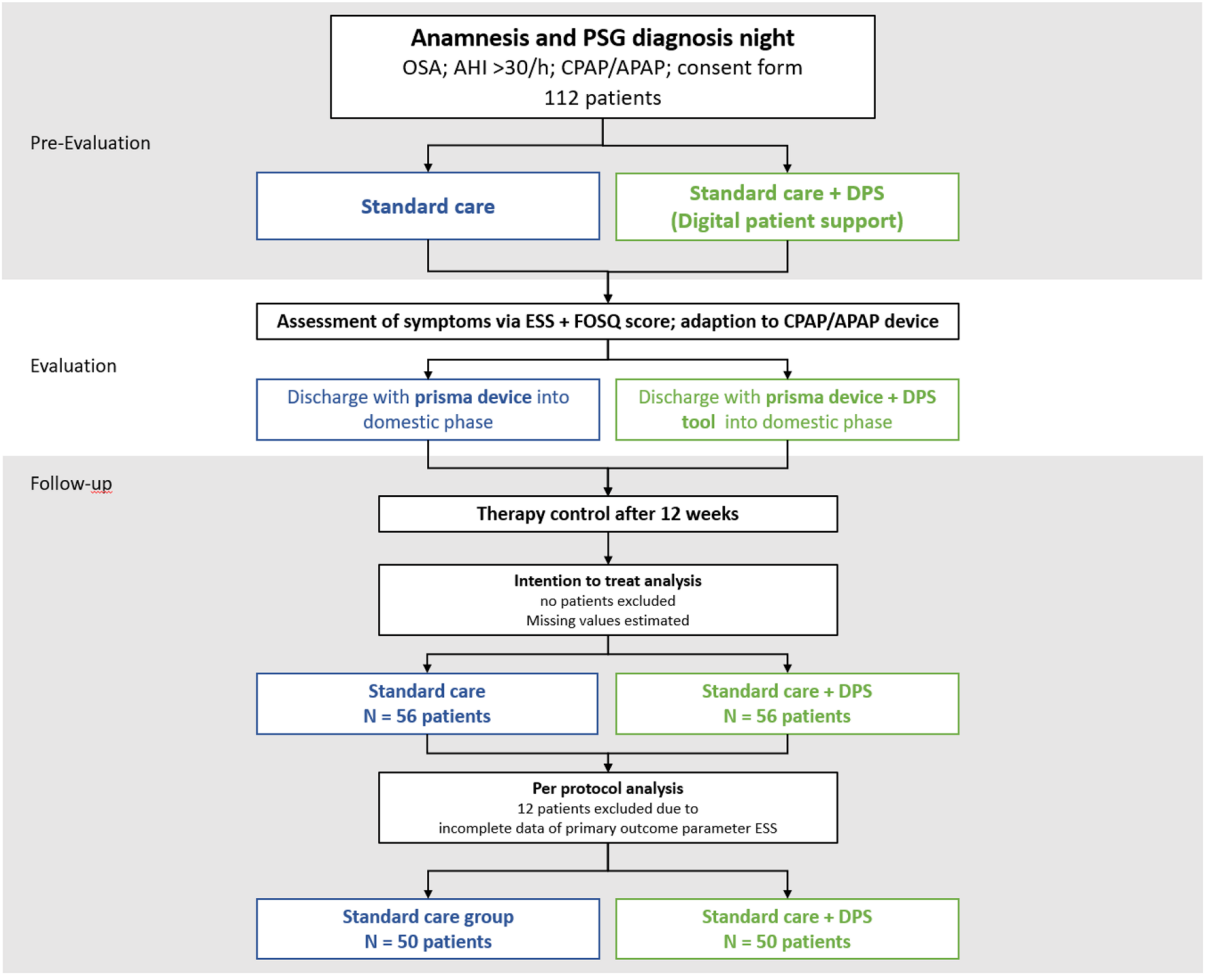
Study flow chart*. OSA* obstructive sleep apnea; *AHI* apnea–hypopnea index; *CPAP* continuous positive airway pressure; *APAP* automatic positive airway pressure *ESS* Epworth Sleepiness Scale; *FOSQ* Functional Outcomes of Sleep Questionnaire

All 130 planned subjects were randomized via an online randomization scheme (randomizer.org) into blocks of 52:78. After undergoing screening and enrollment, participants were assigned to either SC follow-up or to SC + DPS follow-up group, see Fig. 1. As the drop-out rate was lower than estimated and 100 complete data sets were already available after recruitment of 112 patients, the completion of the full number of 130 patients has been waived.

### PAP therapy and interventions

At baseline, all patients received an education session held by a respiratory therapist about OSA and its consequences, proper use and maintenance of the PAP device and mask, and therapy expectations. All patients were provided with a fixed or automatic PAP device (prisma SMART/prisma SOFT, Löwenstein Medical Technology GmbH & Co. KG), a heated humidifier (prismaAQUA, Löwenstein Medical Technology GmbH & Co. KG) if needed, and a fitting interface. The initiation of therapy with anamnesis, diagnosis night, titration night, education, etc., and standardized therapy control after 12 weeks was carried out identically in SC and in SC + DPS group and according to clinical routine. With completion of the study, each patient assessed their satisfaction with the PAP therapy in general and with focus on therapy instructions and support. For this purpose, patients rated their agreement to eight statements on a scale of 0–4 (0: strongly disagree, 1: barely disagree, 2: partly agree, 3: mostly agree, 4: strongly agree).

The following eight statements had to be rated:

1. I was introduced to (auto)CPAP therapy very well.
2. I was introduced to handling of therapy device and equipment very well.
3. I was offered effective ways of self-help and the possibility of personal contact in case of problems.
4. I was guided very well in case of trouble with device handling or therapy itself.
5. I use the (auto)CPAP therapy on a regular basis.
6. The (auto)CPAP therapy supports me. If so (optional): how does the therapy support you?
7. Therapy management is easy for me. If not (optional): what kind of trouble appears?
8. I see no therapy side effects. If not: what kind of side effects do you experience?

The SC + DPS group received electronic therapy support in addition to usual care. The electronic therapy support as a prototype of a mobile application with identical content for feedback and motivation consisted of:

- emails with personalized, schema-guided therapy feedback (derived from device data received via modem or data entered by the patient via electronic questionnaire),
- electronic questionnaires (web-based) on possible problems during therapy and subjective therapy success
- possibility to set personal adherence goals every week,
- links to explanations and videos on therapy and the handling of therapy equipment and accessories,
- provision of data for the trial center in the event of contact by the patient, and for routine therapy monitoring.

Therapy termination did not lead to an analysis exclusion if device usage data was available. Therapy termination was defined as mean device usage < 1 h/day during at least 14 days before therapy control visit.

#### DPS

The DPS was a web-based prototype version of a later mobile app and communicated with the patient via phone or PC. Patients received emails with personalized, automated feedback on their therapy.

The relevant therapy data were received via modem from patients’ therapy device and as information entered by patients via web-based electronic questionnaires. The patient reported outcome enabled the identification of possible problems during therapy and the evaluation of subjective therapy success.

**Fig. 2:**
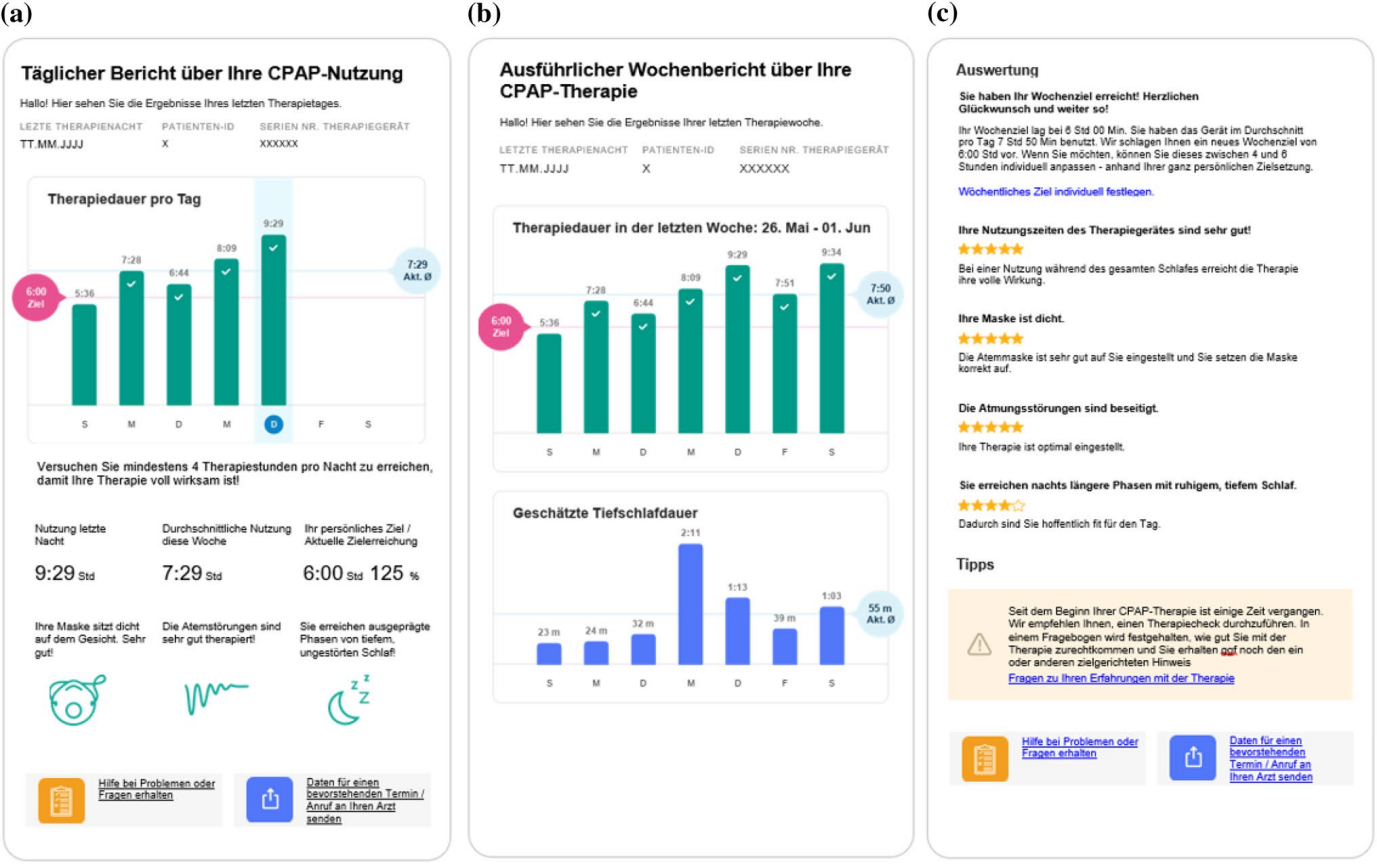
App prototype screens. **a** Daily report: therapy feedback to patient per day with highlighted current day. Therapy duration per day. Advice for therapy duration. Last night usage, mean usage current week, personal goal achievement. Information about mask fitting, therapy efficacy and deep sleep. Links to supportive videos and websites. Link to share a summary of data with the sleep center. **b** Weekly report: therapy feedback to patient per day for the last week. Therapy duration and efficacy for completed week. Estimated duration of deep sleep. **c** Weekly evaluation: detailed information about weekly goal and current goal attainment. Link to set weekly goal. Detailed information about usage, mask fitting, therapy efficacy and deep sleep (with ranking). Hints to improve therapy. Link to therapy check. Links to supportive videos and websites. Link to share a summary of data with the sleep center

The therapy and questionnaire data were combined and analyzed according to a categorization scheme. The results were presented as daily and weekly email reports (see Table 1 and Fig. 2). The weekly report included the possibility to set a personal weekly adherence goal for enhanced therapy commitment (see Fig. 2). As part of the therapy check questionnaire, the DPS also provided links to explanations and videos about therapy and the handling of the therapy device and accessories.

**Table 1:** Feedback scheme

Feedback	Direction of feedback	Description	Number in total during 12 weeks of PAP therapy	Time
Welcome email	DPS → patient	Patient information to their email-based CPAP therapy support	1	Study initiation
Planned therapy check	DPS → patient → DPS	Questionnaire about therapy efficacy, side effects, device and interface including links to explanations and tutorial videos	2	7 days and 4 weeks after study start
Spontaneous therapy check	Patient → DPS		On demand	na
Daily report	DPS → patient	Daily CPAP usage report with possibility to trigger spontaneous therapy check	72	Monday to Saturday
Weekly report	DPS → patient	Detailed weekly CPAP usage report with possibility to trigger spontaneous therapy check and invitation to set adherence goal via link	12	On Sundays
Weekly adherence goal	Patient → DPS	Optional setting of adherence goals	11	On Sundays
Spontaneous summary for experts	Patient → DPS	Summary of device and questionnaire data shared with sleep center as preparation for a visit or phone call	On demand	na
Final summary for experts	Patient → DPS		1	End of patients’ study duration
Farewell	DPS → patient	Credits for participation	1	End of study

The digital patient support (DPS) provided therapy support according to a specific scheme (see Table 1).

### Data assessments/statistical analysis

The aims of the statistical analysis were as follows: (1) comparison between baseline characteristics for the SC group and SC + DPS group; (2) comparison between baseline and follow-up for each group; (3) comparison between groups for each variable; and (4) determination of the validity of each variable. The statistical analysis included descriptive statistics.

Data sets were tested for normal distribution using the Kolmogorov–Smirnov test. Depending on the variance, the data sets were tested for significance using the two-sample *t* test. A *p* value of ≤ 0.05 was considered as statistically significant and was graded as follows: *p ≤ 0.05; **p ≤ 0.01; ***p ≤ 0.001; ****p ≤ 0.0001.

For intention to treat analysis, ESS score was replaced by the value of the one available score (baseline or follow-up visit) if one of the two values was missing. If both ESS Scores were missing, they were replaced by group mean. Missing home therapy device data were replaced by group mean, if follow-up ESS Score was available and by “0” (null), if follow-up ESS Score was not available as therapy was probably discontinued.

All specific analyses were made with complete available data sets and corresponding sample sizes.

The statistical analysis and graphics were created using Microsoft Excel 2016 (Microsoft; Redmond, WA). The data sets generated or analyzed during this study are included in this published article and its supplementary information file. This study was approved by the ethics committee and is registered at NCT05440279.

## Results

As the drop-out rate was lower than estimated the required number of 100 evaluable subjects for per protocol analysis was reached at 112 enrolled patients. 12 patients (SD: 6 vs. SD + DPS: 6) were excluded due to incomplete data (one of the two ESS Scores was missing). An intention to treat analysis regarding ESS Score and adherence to identify a possible selection bias was made for all 112 enrolled patients.

Per protocol, 50 vs. 50 patients were assigned to standard care (SC) group vs. standard care plus digital patient support (SC + DPS) group, while intention to treat patients were distributed 56 vs. 56 to SC group vs. SC + DPS group, see Fig. 1.

Intention to treat (ITT) analysis compared to per protocol (PP) analysis showed similar results regarding ESS and adherence:

total cohort ITT vs. PP: mean ESS baseline 9.1 ± 4.6 vs. 9.3 ± 4.7; mean ESS follow-up 6.7 ± 4.0 vs. 6.6 ± 4.1; mean adherence 305.4 ± 120.1 min. vs. 304.5 min.

SC group ITT vs. PP: mean ESS baseline 9.3 ± 4.1 vs. 9.5 ± 4.2; mean ESS follow-up 7.6 ± 3.9 vs. 7.6 ± 4.1; mean adherence 265.9 ± 120.0 min. vs. 268.8 ± 123.4 min.

SC + DPS group ITT vs. PP: ESS baseline 8.9 ± 5.0 vs. 9.1 ± 5.2; ESS follow-up 5.7 ± 3.9 vs. 5.5 ± 3.9; mean adherence 344.2 ± 106.9 vs. 338.8 ± 107.9 min.

Per protocol analysis revealed the following:

No difference was found in characteristics of SC vs. SC + DPS for age, initial diagnostic apnea–hypopnea index, BMI, and ESS baseline score, see Table 2. Of the 100 patients, 97 used continuous PAP and 3 used automatic PAP therapy mode. For follow-up results, see Table 3.

**Table 2:** Demographic and clinical data of the study population—baseline

	Total cohort	Standard care	Standard care with digital patient support	*p* Value
Subjects (*n*)	100	50	50	–
Demografics				
Age in years (mean ± SD)	52.8 ± 11.6	53.9 ± 10.8	51.7 ± 12.3	0.174
Sex				
Male (*n*)	76	38	38	–
Female (*n*)	24	12	12	–
BMI in kg/m^2^ (Mean ± SD)	33.7 ± 5.7	33.8 ± 6.7	33.5 ± 4.5	0.387
Diagnostic PSG				
AHI (n/h)				
Mean ± SD	51.0 ± 16.6	51.1 ± 15.5	50.9 ± 17.7	0.476
Median (IQR)	48.4 (24.4)	48.5 (26.1)	48.1 (20.9)	–
oAI (n/h)				
Mean ± SD	25.8 ± 21	26.9 ± 24.2	24.8 ± 17.7	0.310
Median (IQR)	19.6 (25.0)	17.8 (24.5)	21.8 (24.5)	–
Questionnaire scores				
ESS—baseline				
Mean ± SD	9.3 ± 4.7	9.5 ± 4.2	9.1 ± 5.2	0.315
Median (IQR)	9.0 (6.25)	9.0 (6.5)	8.5 (7.0)	–
FOSQ—baseline				
Mean ± SD	16.4 ± 2.5	16.2 ± 2.6	16.7 ± 2.4	0.284
Median (IQR)	16.8 (4.1)	16.7 (4.5)	16.9 (3.7)	–

Data are presented as mean ± standard deviation or median (interquartile range, IQR), unless otherwise stated. *BMI* body mass index; *PSG* polysomnography; *AHI* apnea–hypopnea index; *oAI* obstructive apnea-index; *ESS* Epworth Sleepiness Scale; *FOSQ* Functional Outcomes of Sleep Questionnaire; *p* value refers to comparison of standard care group and standard care with digital patient support group

**Table 3:** Summary of findings for outcomes

	Total cohort (*n* = 100)	SC (*n* = 50)	SC + DPS (*n* = 50)	*p* value
Questionnaire scores				
ESS—baseline				
Mean ± SD	9.3 ± 4.7	9.5 ± 4.2	9.1 ± 5.2	0.315
Median (IQR)	9.0 (6.3)	9.0 (6.5)	8.5 (7.0)	
ESS—follow-up				
Mean ± SD	6.6 ± 4.1	7.6 ± 4.1	5.5 ± 3.9	0.006**
Median (IQR)	6.0 (6.3)	6.5 (7.8)	5.0 (4.8)	
FOSQ—baseline				
Mean ± SD	16.4 ± 2.5	16.2 ± 2.6	16.7 ± 2.4	0.284
Median (IQR)	16.8 (4.1)	16.7 (4.5)	16.9 (3.7)	
FOSQ—follow-up				
Mean ± SD	18.2 ± 2.1	17.8 ± 2.2	18.5 ± 1.9	0.053
Median (IQR)	19.1 (2.6)	18.5 (2.6)	19.3 (1.3)	

	Total cohort (*n* = 98)	SC (*n* = 48)	SC + DPS (*n* = 50)	*p* value
Adherence (Ø min/d)				
Mean ± SD	304.5 ± 120.4	268.7 ± 122.1	338.8 ± 106.8	0.002**
Median (IQR)	327.1 (146.1)	305.3 (166.8)	357.9 (108.0)	
Adherence (d)				
Mean ± SD	57.9 ± 24.5	50.8 ± 25.7	64.6 ± 21.1	0.002**
Median (IQR)	67.0 (31.5)	59.5 (45.3)	70.0 (19.5)	
Duration of stable respiration (min)				
Mean ± SD	75.7 ± 50.7	64.3 ± 41.2	79.7 ± 56.6	0.015*
Median (IQR)	70.1 (76.1)	61.4 (65.4)	79.7 (109.9)	
Pressure P50 (hPa)				
Mean ± SD	10.2 ± 1.5	10.4 ± 1.5	10.1 ± 1.6	0.164
Median (IQR)	10.3 (1.6)	10.3 (2.0)	10.2 (2.0)	
Leakage (l/min)				
Leakage P50				
Mean ± SD	4.0 ± 3.6	4.6 ± 4.0	3.4 ± 3.2	0.055
Median (IQR)	2.8 (3.7)	3.5 (5.1)	2.4 (3.0)	
Leakage P95				
Mean ± SD	18.7 ± 11.7	20.6 ± 12.4	16.9 ± 10.7	0.061
Median (IQR)	15.8 (18.0)	18 (20.7)	13 (16.8)	
Apnea indices (n/h)				
AHI—baseline (PSG)				
Mean ± SD	51.0 ± 16.6	51.1 ± 15.5	50.1 ± 17.7	0.476
Median (IQR)	48.4 (24.4)	48.5 (26.1)	48.1 (20.9)	
AHI—home therapy (device)				
Mean ± SD	3.8 ± 2.8	4.1 ± 3.0	3.5 ± 2.6	0.170
Median (IQR)	2.9 (3.5)	3.1 (3.3)	2.8 (3.5)	
oAI—baseline				
Mean ± SD	25.8 ± 21.0	26.9 ± 24.2	24.8 ± 17.4	0.310
Median (IQR)	19.6 (25.0)	17.8 (24.5)	21.8 (24.5)	
oAI—home therapy (device)				
Mean ± SD	0.9 ± 0.9	1.0 ± 1.0	0.8 ± 0.8	0.147
Median (IQR)	0.6 (1.1)	0.6 (1.1)	0.5 (1.1)	

	Total cohort (*n* = 97) PSG/PG = 55/43	SC (*n* = 48) PSG/PG = 27/21	SC + DPS (*n* = 49) PSG/PG = 28/21	*p* value
AHI—follow-up (PSG/PG)				
Mean ± SD	5.7 ± 5.6	5.8 ± 5.4	5.6 ± 5.9	0.432
Median (IQR)	4.2 (5.0)	4.5 (5.8)	4.1 (4.8)	
oAI—follow-up (PSG/PG)				
Mean ± SD	0.7 ± 1.8	0.6 ± 0.9	0.9 ± 2.4	0.236
Median (IQR)	0.2 (0.8)	0.2 (0.9)	0.2 (0.6)	

	Total cohort (*n* = 99)	SC (*n* = 50)	SC + DPS (*n* = 49)	*p* value
Patient satisfaction				
Median (IQR)	3.7 (0.6)	3.6 (0.6)	3.7 (0.5)	0.048*

Data are presented as mean ± standard deviation or median (interquartile range, IQR), unless otherwise stated. *ESS* Epworth Sleepiness Scale; *FOSQ* Functional Outcomes of Sleep Questionnaire; *P 50* Median; *P 95* 95th percentile; *AHI* apnea–hypopnea index; *oAI* obstructive apnea-index

*p* value refers to standard care group and standard care with digital patient support group. *p ≤ 0.05; **p ≤ 0.01

### ESS scale and adherence

**Fig. 3:**
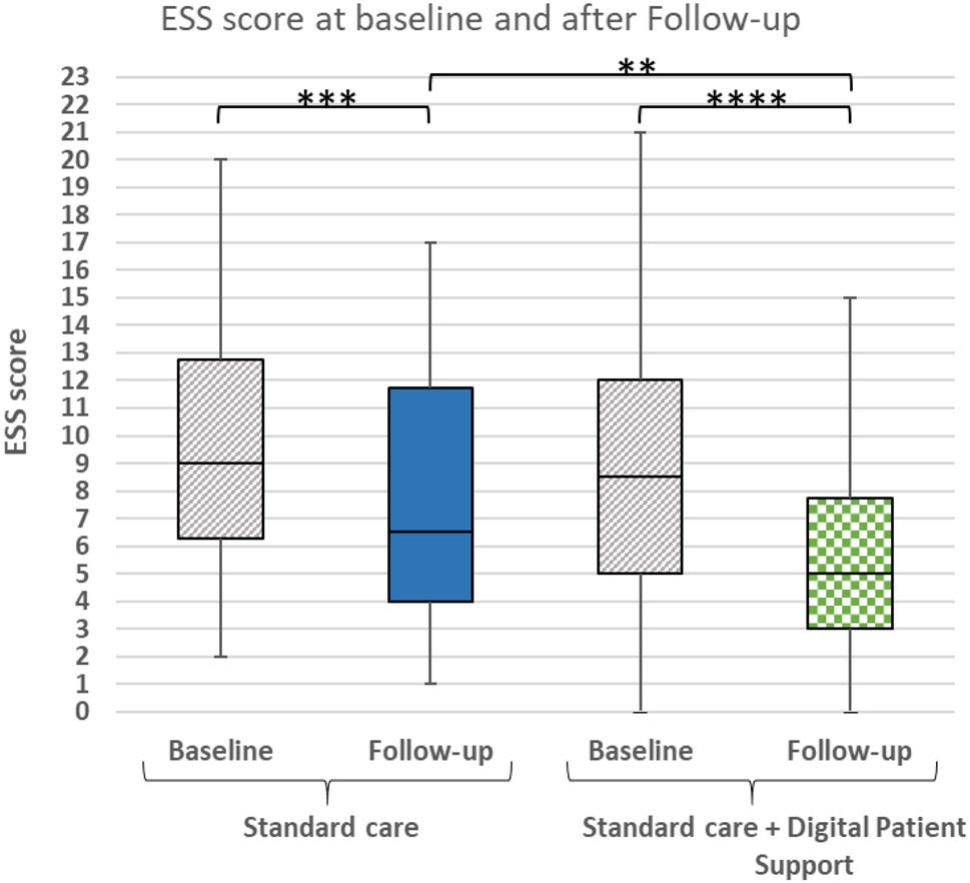
Box plots illustrating ESS score at baseline and follow-up per study arm. Horizontal line within boxes depicts the median. Upper and lower box boundaries are 75th and 25th percentile, respectively. **p ≤ 0.01; ***p ≤ 0.001; ****p ≤ 0.0001

ESS score compared at baseline and follow-up of the total cohort significantly improved (9.3 ± 4.7 vs. 6.6 ± 4.1; *p* < 0.0001), see Fig. 3. In addition, the reduction of the ESS score was significantly higher by 1.7 score points in the SC + DPS group compared to standard care group after 12 weeks, see Table 3.

A subgroup of 36 patients (20 SC vs. 16 SC + DPS) with excessive daytime sleepiness (ESS > 10) at baseline showed a difference between SC and SC + DPS group regarding improvement of ESS after 12 weeks in favor of SC + DPS group: ESS Score SC group baseline 13.8 ± 2.6 and follow-up 9.7 ± 3.8 vs. ESS Score SC + DPS group baseline 15.3 ± 2.8 and follow-up 7.7 ± 4,6. Remaining high ESS Score > 10 at follow-up visit was documented for 10 out of 20 patients (50%) of the SC group vs. 5 out of 16 patients (31.2%) of the SC + DPS group.

**Fig. 4:**
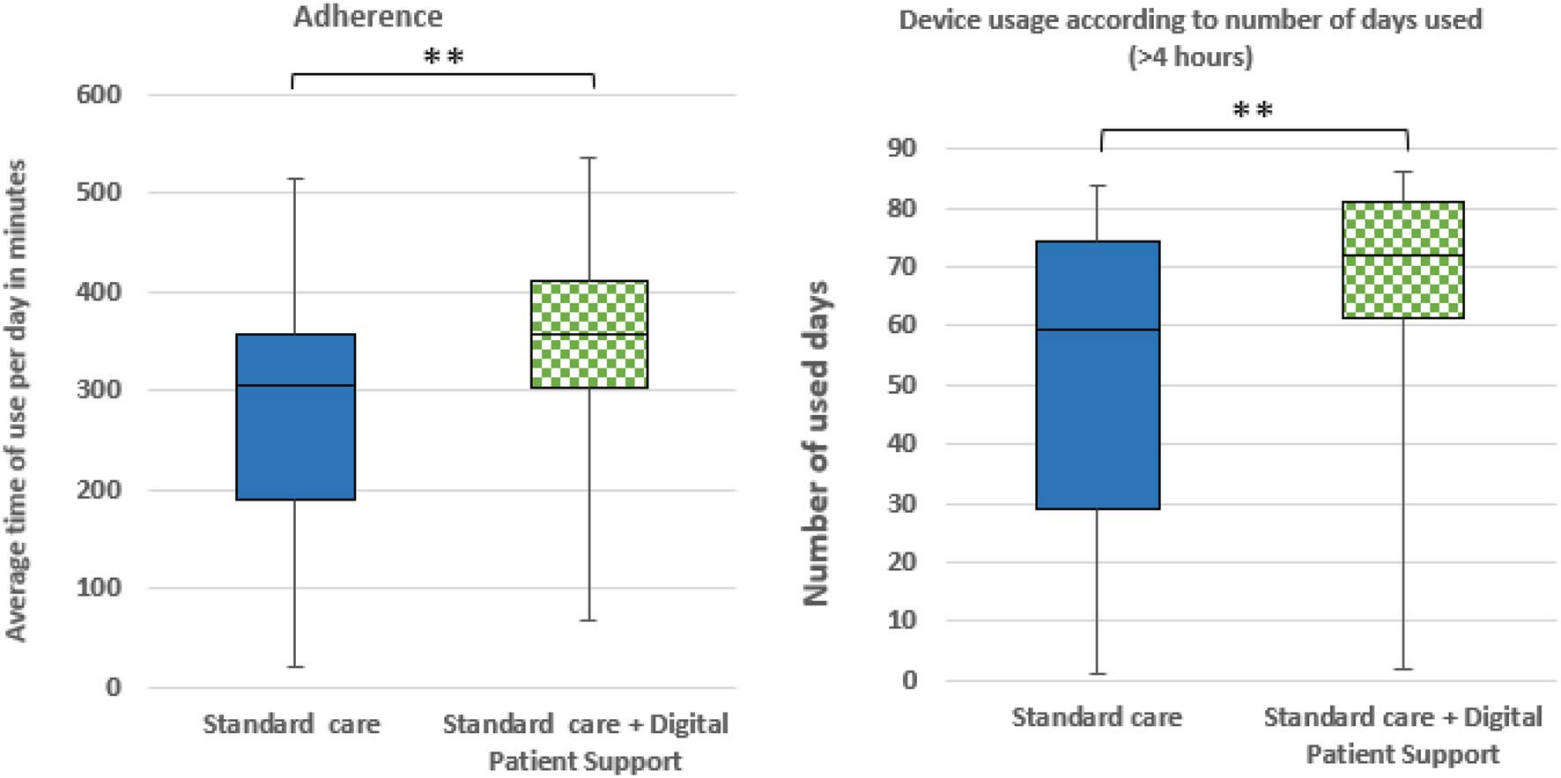
Box plots illustrating adherence and device usage according to number of used days (> 4 h) after 12 weeks per study arm. Horizontal line within boxes depicts the median. Upper and lower box boundaries are 75th and 25th percentile, respectively. **p ≤ 0.01

Two patients of the SC group had to be excluded from adherence analysis because of missing therapy device data. After 12 weeks, in the cohort of 98 patients (48 SC vs. 50 SC + DPS) mean PAP adherence was significantly higher in the SC + DPS group compared to standard care group with a mean difference of 70.1 min (SC 268.7 ± 122.1 vs. SC + DPS 338.8 ± 106.8; *p* = 0.002), see Fig. 4. Moreover, device usage measured by number of days with > 4 h usage was significantly higher in the SC + DPS group compared to standard care group, see Fig. 4.

Applying the definition of therapy termination (defined as mean device usage < 1 h/day) to the adherence analysis (cohort of 98 patients), seven patients in the SC group and only three patients in the SC + DPS group (10 in total) would be classified as having discontinued therapy.

Our data analysis shows that for this specific study cohort a threshold of 299 min for average adherence during the last 29 days prior to follow-up ESS assessment could achieve specificity and sensitivity of 0.64 each to predict residual daytime sleepiness (ESS follow-up ≥ 10). No significant bivariate correlation could be found between adherence and follow-up ESS.

### FOSQ scale

The comparison of baseline and follow-up values of the total cohort also shows a significant increase in the FOSQ score (16.4 ± 2.5 vs. 18.2 ± 2.1; *p* < 0.0001), see Table 3. After 12 weeks, the digital patient support group indicates a trend towards a better FOSQ score compared to standard care group.

### AHI, pressure, leakage, and duration of stable respiration (indication for undisturbed sleep)

The residual AHI was detected based on the device recordings during the 12 weeks of home therapy as well as on the sleep center follow-up visit including PG or PSG measurement after 12 weeks of home therapy. The device AHI relates to the adherence analysis cohort of 98 patients with a distribution of 48 (SC) vs. 50 (SC + DPS), while the AHI measured by the sleep lab relates to a cohort of 97 patients with a distribution of 48 (SC) vs. 49 (SC + DPS) due to missing therapy control night data of one of the 98 adherence analysis cohort. In both cohorts, no difference was found between the SC and SC + DPS group.

Therapy pressure, leakage and duration of stable respiration were determined based on the mean values recorded by the therapy device during the 12 weeks of home therapy.

After 12 weeks, in the cohort of 98 patients (48 SC vs. 50 SC + DPS) no difference was found in the applied pressure between both groups, see Table 3. However, with a difference of 3.7 l/min (*p* = 0.061) SC + DPS group showed a trend towards lower P95 (95th percentile) of leakage compared to SC group and significantly longer periods of stable respiration, serving as a potential indicator of physically restorative deep sleep.

### Interventions/contacts with the provider or sleep lab

In view of the participants, 35 patients in total (15 of SC vs. 20 of SC + DPS group) had contact with the provider. Mean compliance in this subgroup was 306.1 ± 126.9 compared to 304.5 ± 120.4 in the total cohort.

With reference to the number of contacts, standard care group has made contact 18 times (corresponds to an average of 0.36 per patient), whereas standard care + digital patient support group has made contact 29 times (average of 0.58 per patient) with the provider or sleep center during study duration.

A distinction between the types of contact was made between a telephone call and an on-site appointment. For standard care group 10 phone calls and 9 on-site appointments were recorded. Standard care + digital patient support group made 23 phone calls and arranged 7 on-site appointments.

A more detailed evaluation of the reasons for contact is shown in Table 4. As patients could have had more than one reason for contacting their provider or sleep center, the total numbers in Table 4 are different from the mentioned numbers of contacts. In the SC + DPS group, 12 contacts by nine patients were made due to the additional digital support tool and could only appear in this study arm (“welcome email not received” or “problems with data transmission by modem”).

**Table 4:** Reasons and number of phone contacts or on-site appointments per study arm

Reason for contact	Total	SC group	SC + DPS group
DPS-specific feedback			
Welcome e-mail not received	8	–	8
Problems with data transmission by modem	4	–	4
Therapy/equipment feedback			
Feedback on therapy use/missing therapy benefit	9	1	8
Mask problems/desire to change mask	25	14	11
Malfunction of therapy device	1	–	1
Problems with therapy pressure/therapy mode	7	5	2
Problems with circuit system	2	1	1
Side-effects			
Dry mouth/nose	8	3	5
Aerophagy	1	–	1
Nasal congestion/rhinorrhea	4	4	–
Feeling of suffocation	1	1	–
Dyspnea	1	1	–
Other			
Seizure	1	1	–
Total	72	31	41

Several reasons for contact are possible per patient

*SC* standard care; *DPS* digital patient support.

The most frequently reported reasons other than the SC + DPS group specific were mask problems/desire to change mask, dry mouth and feedback on therapy use/missing therapy benefit.

### Patient satisfaction

One patient of the SC + DPS group was excluded from analysis of patient satisfaction because of missing data. The assessment of patient satisfaction was determined by means of eight statements, rated on a scale of 0–4, with a 4 being the best rating. We calculated a statistically significant difference between SC + DPS and SC group (0.1 points, *p* = 0,048). As therapy satisfaction was very high in both groups (see Table 3) we do not consider this difference as clinically meaningful.

## Discussion

### ESS score (primary endpoint) and adherence

To our knowledge, this is one of the first randomized controlled trials showing that daytime sleepiness improved by additional 1.7 score points (to follow-up ESS values of 5.5 ± 3.9 in −SC + DPS vs. 7.6 ± 4.1 in SC) and (auto)CPAP therapy adherence increased by additional 70.1 min/d (338.8 ± 106.8 in SC + DPS vs. 268.7 ± 122.1 in SC) significantly and to a clinically relevant extend in patients with severe OSA when using a digital patient support tool in combination with an (auto)CPAP device compared to standard care alone after 12 weeks. Though other studies such as Malhotra et. al. [32] recruited a large sample size, their evidence level is low as they are not randomized controlled. The difference between the two groups illustrates an area between two health conditions (lower normal vs. higher normal daytime sleepiness) that might make a difference for a single patient`s well-being. Especially patients with an ESS Score > 10 at baseline are at risk to fail in normalizing the ESS Score during the first 12 weeks of therapy [30]. Additional improvement of daytime sleepiness through the digital patient support might have a significant positive effect on patients’ daily life like improved concentration, the experience of alertness or being less sleepy while driving. Therefore, it has the potential to strengthen the therapy effect in the decisive initial therapy phase.

Obtaining a frequent and long nightly PAP device use is mandatory to achieve clinical benefits by PAP therapy, such as enhanced functional outcome, reduction in blood pressure, or improvement in memory [3–6]. Our hypothesis that a digital patient support tool would improve adherence was confirmed and is in line with previous investigations [22]. With the prototype of the mobile app, even an increased improvement could be achieved.

The impact of telemonitoring on improvement of adherence was already demonstrated in former studies: Aardoom et al. found in a meta-analytic review of 18 RCTs with eHealth interventions that on average the improvement in therapy adherence was about 0.5 h [22]. Bouloukaki et al. [33], showed a significant improvement of 90 min/d CPAP usage and 3 points in ESS score after 24 months as a result of additional visits, telephone calls and education in 3100 OSA patients. Hwang et al. showed a significant effect on 90-day CPAP use in a 4-arm randomized controlled trial with 1455 OSA patients of 60 min/d after 90 days with a web-based OSA education and automated patient feedback in addition to standard care [19]. However, telemedicine-based patient education alone resulted in no significant adherence improvement. Furthermore, no differences in ESS or the FOSQ-10 score in any intervention study arm compared to standard care was observed. Bouloukaki et al. [33] concluded that additional video education session (15 min), a lecture from the sleep clinic’s registered nurses (10–15 min), phone calls by the nurse on the 2nd and 7th day to discuss any concerns, followed by home visits in case of concerns, two additional reviews by the sleep specialist, invitations to discuss therapy barriers, experiences, concerns, fears and beliefs, etc. is a time-consuming care beyond measure and difficult to integrate into clinical routine, the author discloses that the “intensive intervention entailed an additional cost of 30% above the cost of the standard intervention” [33]. Munafo et al. [34] showed in a prospective study that a web-based automated telehealth-messaging program reduced the time expenditure for healthcare professionals by 59%, compared to standard care, with maintaining adherence and effectiveness.

Considering former studies and our current outcomes concerning the impact of telemonitoring on (auto)CPAP therapy results, the use of the app prototype alone does not constitute the reason for altered ESS and FOSQ scores in our study results. Rather, the improvement is a consequence of increased (auto)CPAP use. As Antic et al. [30] already confirmed, indicators for subjective daytime sleepiness, functional status, and sleep-related quality of life, such as the ESS and FOSQ scores, are dose-dependent on CPAP treatment, with greater improvements in more-adherent patients. Regarding a presumable relation of (auto)CPAP adherence and ESS score, our results show an association of (auto)CPAP adherence with a reduction of ESS Score and are thereby in line with the meta-analysis of Li et al. [35]. However, the impact of factors such as age, BMI, baseline ESS, etc. is at least equally strong. Therefore, a prediction of residual daily sleepiness would have to include several factors and the identified discrimination threshold of less than 299 min adherence as predictor for residual sleepiness is not likely to be transferrable to patient cohorts with different characteristics. To obtain significant results regarding the correlation between adherence and follow-up ESS, a larger sample size is needed.

The significantly improved ESS and the trend in the FOSQ score compared to standard care in our study indicates that additional digital patient support has a positive impact on daytime sleepiness and functional status in adults. This might be reducible to our carefully designed automated feedback that considered OSA patient characteristics (well-documented in the thesis “PSI theory and adherence in nightly PAP therapy” of the psychology student M. Michalzyk) and the impact of the possibility to set individual and reachable personal goals on human decisions [36]. In addition, the personal contact patients made during home therapy phase might have affected their outcome. When looking at the number of phone contacts or on-site appointments, the group using the app prototype contacted the sleep therapist more frequently. However, considerably less than 1 contact per patient was registered on average and 12 of those contacts (made by 9 patients) are referrable to the study design or immature technology of the prototype as participants did not receive the welcome email or reported problems with data transmission by modem. This even might have led to frustration during the early therapy experience of these patients but apparently not to a degree of significant negative impact on their therapy adherence. However, initial technical difficulties cannot be generally ruled out in the future. Nevertheless, problems with data transmission by modem become obsolete by providing the possibility to use Bluetooth. A missing welcome email becomes irrelevant outside the study, while installation problems still could pose a small risk. The more frequent contact of the SC + DPS group even might have had a positive influence on these patients` adherence due to personal interaction with healthcare professionals and a possibly related motivating effect. In addition, the experience of solving a problem-like getting transmission of data started might have had an inspiring influence on SC + DPS group. As contacts due to therapy use or missing therapy benefit were mainly made by the SC + DPS group, the examination of the automated therapy feedback might have been the more relevant factor than the contact itself. Adherence of the subgroup with contacting patients is with less than 2 min not higher than in the total cohort (306.1 ± 126.9 compared to 304.5 ± 120.4) and over two-thirds of the population had no personal contacts at all. No difference was found in age, BMI, diagnosed AHI and oAI and in baseline ESS and FOSQ score between both groups. In addition, follow-up data shows no difference in the applied therapy pressure. Therefore, it is far from likely, that either the number of personal contacts nor other patient-related factors are the cause for increased adherence and possibly related improvement of ESS in the SC + DPS group.

The trend of lower leakage is due to the app use, as the application is designed to monitor and support the user’s therapy on a daily basis by providing personalized, schema-guided feedback, electronic questionnaires on potential problems and links to explanations and videos on therapy and the handling of therapy equipment. Hence, automated feedback on acute mask leakage may help the patient to check and improve mask fit or suggest a mask change. It is, therefore, no surprise that more than twice as many responses regarding mask problems were recorded in the standard care group. With the background, that CPAP pressure can be associated with an increased risk of unintentional leakage [37], it needs to be mentioned that in our study no difference between both groups was found in the used therapy pressure after 12 week follow-up.

No difference in the AHI/oAI follow-up was identified between SC and SC + DPS. The cause might be that both groups were already well-adjusted during titration night and no readjustment by the sleep center had to be recommended for the SC + DPS group by the app prototype.

Furthermore, a mean residual AHI of 4.1 n/h in the standard care group indicates how effectively the (auto)CPAP therapy devices used in this trial treat sleep apnea in clinical routine without additional digital support. The clinical benefit of those therapy devices even without additional DPS is also clearly recognizable as after 12 week adherence is high and ESS and FOSQ score improved significantly, even though patients showed only moderate daytime sleepiness at baseline. Furthermore, therapy termination rate was particularly low in both study arms which might be the result of the high level of care provided which is confirmed by patient satisfaction with the PAP therapy in general and with focus on therapy instructions and support.

Personal digital support in terms of an email-based mobile app prototype was well-accepted by PAP patients and worked reliably. In addition, the DPS was consistently used as a supportive tool and not as a replacement of conventional therapy support, as all patients continued standard therapy care while using the DPS.

In comparison with previous reports like Munafo et al. [34] or Malhotra et. al. [32], our study shows better improvement in adherence and consequent reduction in daytime sleepiness. We found no difference in age, BMI, diagnosed AHI and baseline ESS score compared to these reports. The better results might be due to a more intuitive or attractive handling of the tool. In addition, differences in comorbidities, which were not particularly investigated in this study, might be a reason for the success of our digital patient support tool.

### Study strengths

All subjects followed the identical initiation of therapy and standardized therapy follow-up procedure according to clinical routine. SD and SD + DPS group had the same education session held by a respiratory therapist about OSA and its consequences, proper use and maintenance of the PAP device, mask fitting, and therapy and study expectations. All patients were provided with the same (auto)CPAP device types.

This study was designed as a prospective, randomized and controlled trial. After the enrollment, participants were randomized to either SC or to SC + DPS group. Consideration of the baseline data shows that randomization was successful. No difference was found in age, BMI, diagnosed AHI and oAI and in ESS and FOSQ score between both groups. In addition, follow-up data shows no difference in the applied therapy pressure. The drop-out rate was particularly low, as only six subjects in the SC and six in the SC + DPS group were excluded from the study, what most probably is a result of the excellent care and education provided by the sleep lab and homecare provider personnel. This is also reflected by generally low therapy termination rates and high adherence values in both groups, posing a significant challenge to the DPS tool to show an additional positive effect.

Through our eyes the homogeneity of the groups concerning demographic, baseline and follow-up data are seen as a huge study strength as none of the factors mentioned above can be the cause of the significant differences in adherence, ESS score, and duration of stable respiration between groups. In fact, the automated feedback seems to have a strong impact on these parameters.

Malhotra et al. [32] showed the potential contribution of a patient management app (Res Med “My Air”) to improve adherence, using big data and propensity score matching. This was not a randomized study, and thus prone to possible selection bias; those who were highly motivated to treat with a high level of education might have used this application selectively. Our current study, despite being small in scale, has the strength of being randomized, which clears this bias.

Munafo et al. [34] showed that a web-based automated telehealth-messaging program involving healthcare providers to manage patients reduced the time expenditure by 59% with similar results in adherence and effectiveness compared to standard care. To our knowledge no randomized controlled trials exist that show improved adherence and daytime sleepiness based on an only patient-managed automated coaching tool compared to standard care like our current trial. The automated monitoring and feedback of sleep status, identification of problems and presentation of solutions lead to patient empowerment and psychological reinforcement.

### Study limitations

The current investigation had limitations that must be considered.

First, the cohort of participants consisted of patients diagnosed and treated in the same center and with severe OSA only. Hence, the transferability of the study results to patients with mild or medium OSA is limited. Participants were predominantly male and obese, which also impedes the transferability to other patient groups. However, distribution of sex and BMI is in line with those in similar studies [16, 38–40] indicating that the patient population in the current study is representative for the use of telemedical support. Furthermore, the distribution of sex within the two groups was identical (12 women and 38 men each).

Second, the observation period of the study was only 12 weeks, although the fact that adherence decreases within the first months is well-known [8, 10, 41]. However, there is no evidence that the change in adherence of the two groups would differ over a longer period of time.

Third, time expenditure per patient and per study arm was not determined. However, in the staff's assessment, which was verbally communicated, the SC + DPS group did not generate a higher time expenditure compared to standard care group. Evidence is the number of phone contacts or on-site appointments. If the feedback due to technical problems with the app prototype is not taken into account, the PAP therapy of the SC + DPS group has generated about the same amount of follow-up contacts. Nevertheless, it is impossible to distinguish how much time the individual study groups generated per response.

Another limitation is that the unknown influence of home environments (e. g. relatives helping with setup/operation of the app). This was not considered in previous studies and is difficult to measure.

## Conclusion

Digital feedback and support as incorporated in the app prototype and the later mobile app may enable patients to improve their adherence and long-term outcome of (auto)CPAP therapy by feedback, motivation and troubleshooting support. Expert interventions can be focused on patients who cannot succeed autonomously, e. g. in case of an elevated residual AHI or residual sleepiness. In the future, receiving therapy device data and self-reported outcome may support experts with intervention decisions such as summoning patients for an in-lab therapy adjustment. Technologies such as a digital patient support tool bear the potential to enhance additional clinical benefits associated with PAP treatment, such as reduced blood pressure. Further randomized controlled trials are needed to evaluate the current results in greater detail.

### Summary

In summary, this prospective, randomized controlled study indicates that the addition of a digital patient support tool for therapy monitoring and motivation of PAP-naïve patients with severe OSA resulted in improved (auto)CPAP adherence and daytime sleepiness after 12 weeks compared to standard care. The app prototype was well-accepted by those patients and worked reliably in combination with the (auto)CPAP therapy devices.

### Supplementary Information

Below is the link to the electronic supplementary material.

Supplementary file1 (XLSX 40 KB)

## Abbreviations

AHI: Apnea–hypopnea index
(auto)CPAP: Automatic continuous positive airway pressure
CPAP: Continuous positive airway pressure
ESS: Epworth Sleepiness Scale
FOSQ: Functional Outcomes of Sleep Questionnaire
OSA: Obstructive sleep apnea
PAP: Positive airway pressure
PSG: Polysomnography
SC: Standard care
DPS: Digital patient support

## References

[1] Duran J, Esnaola S, Rubio R, Iztueta A (2001). Obstructive sleep apnea-hypopnea and related clinical features in a population based sample of subjects aged 30 to70 yr. Am J Respir Crit Care Med.

[2] Peppard PE, Young T, Barnet JH, Palta H, Hagen EW, Hla KM (2013). Increased prevalence of sleep-disordered breathing in adults. Am J Epidemiol.

[3] Weaver TE, Maislin G, Dinges DF (2007). Relationship between hours of CPAP use and achieving normal levels of sleepiness and daytime functioning. Sleep.

[4] Engleman HM, Martin SE, Deary IJ, Douglas NJ (1997). Effect of CPAP therapy on daytime function in patients with mild sleep apnoea/hypopnoea syndrome. Thorax.

[5] Centers for Medicare & Medicaid Services. Continuous and bi-level positive airway pressure (CPAP/BPAP) devices: complying with documentation and coverage requirements. Available from: http://www.cms.gov/Outreach-and-Education/Medicare-Learning-Network-MLN/MLNProducts/downloads/PAP_DocCvg_Factsheet_ICN905064.pdf.%20Accessed%2025%20Oct%202021. Accessed 25 Oct 2021.

[6] Barbe F, Duran-Cantolla J, Capote F (2010). Long-term effect of continuous positive airway pressure in hypertensive patients with sleep apnea. Am J Respir Crit Care Med.

[7] Peppard PE, Young T, Barnet JH, Palta H, Hagen EW, Hla KM (2013) Increased prevalence of sleep-disordered breathing in adults. Am J Epidemiol 177(9):1006–1014. 10.1093/aje/kws342. 10.1093/aje/kws342 PMC3639722 23589584

[8] Stepnowsky CJ, Moore PJ (2003). Nasal CPAP treatment for obstructive sleep apnea: developing a new perspective on dosing strategies and compliance. J Psychosom Res.

[9] Sin DD, Mayers I, Man GC (2002). Long-term compliance rates to continuous positive airway pressure in obstructive sleep apnea: a population-based study. Chest.

[10] Budhiraja R, Parthasarathy S, Drake CL (2007). Early CPAP use identifies subsequent adherence to CPAP therapy. Sleep.

[11] Billings ME, Auckley D, Benca R (2011). Race and residential socioeconomics as predictors of CPAP adherence. Sleep.

[12] Platt AB, Field SH, Asch DA (2009). Neighborhood of residence is associated with daily adherence to CPAP therapy. Sleep.

[13] Wild MR, Engleman HM, Douglas NJ, Espie CA (2004). Can psychological factors help us to determine adherence to CPAP? A prospective study. Eur Respir J.

[14] Means MK, Edinger JD, Husain AM (2004). CPAP compliance in sleep apnea patients with and without laboratory CPAP titration. Sleep Breath.

[15] Borel JC, Tamisier R, Dias-Domingos S (2013). Type of mask may impact on continuous positive airway pressure adherence in apneic patients. PLoS ONE.

[16] Sparrow D, Aloia M, Demolles DA, Gottlieb DJ (2010). A telemedicine intervention to improve adherence to continuous positive airway pressure: a randomized controlled trial. Thorax.

[17] Chai-Coetzer CL, Luo YM, Antic NA (2013). Predictors of long-term adherence to continuous positive airway pressure therapy in patients with obstructive sleep apnea and cardiovascular disease in the SAVE study. Sleep.

[18] Dzierzewski JM, Wallace DM, Wohlgemuth WK (2016). Adherence to continuous positive airway pressure in existing users: self-efficacy enhances the association between continuous positive airway pressure and adherence. J Clin Sleep Med.

[19] Hwang D, Chang JW, Benjafield AV, Crocker ME, Kelly C, Becker KA, Kim JB, Woodrum RR, Liang J, Derose SF (2018). Effect of telemedicine education and telemonitoring on CPAP adherence: the Tele-OSA randomized trial. Am J Respir Crit Care Med.

[20] Richter M, Schroeder M, Domanski U, Schwaibold M, Nilius G (2022). Reliability of respiratory event detection with continuous positive airway pressure in moderate to severe obstructive sleep apnea—comparison of polysomnography with a device-based analysis. Sleep and Breath.

[21] Lettieri CJ, Williams SG, Collen JF, Wickwire EM (2017). Treatment of obstructive sleep apnea. Achieving adherence to positive airway pressure treatment and dealing with complications. Sleep Med Clin.

[22] Aardoom JJ, Loheide-Niesmann L, Ossebaard HC, Riper H (2020). Effectiveness of eHealth interventions in improving treatment adherence for adults with obstructive sleep apnea. Meta-analytic review. J Med Internet Res.

[23] Sawyer AM, Gooneratne NS, Marcus CL, Ofer D, Richards KC, Weaver TE (2011). A systematic review of CPAP adherence across age groups: clinical and empiric insights for developing CPAP adherence interventions. Sleep Med Rev.

[24] Pépin JL, Tamisier R, Hwang D, Mereddy S, Parthasarathy S (2017). Does remote monitoring change OSA management and CPAP adherence?. Respirology.

[25] Nilius G, Schroeder M, Schoebel C (2021). Telehealth and its implementation in respiratory sleep medicine. Curr Opin Pulm Med.

[26] Johns MW (1991). A new method for measuring daytime sleepiness: the Epworth sleepiness scale. Sleep.

[27] Weaver TE, Laizner AM, Evans LK (1997). An instrument to measure functional status outcomes for disorders of excessive sleepiness. Sleep.

[28] Weaver TE, Mancini C, Maislin G (2012). Continuous positive airway pressure treatment of sleepy patients with milder obstructive sleep apnea: results of the CPAP Apnea Trial North American Program (CATNAP) randomized clinical trial. Am J Respir Crit Care Med.

[29] Patel SR (2003). Continuous positive airway pressure therapy for treating sleepiness in a diverse population with obstructive sleep apnea: results of a meta-analysis. Arch Intern Med.

[30] Antic NA, Catcheside P, Buchan C, Hensley M, Naughton MT, Rowland S (2011). The effect of CPAP in normalizing daytime sleepiness, quality of life and neurocognitive function in moderate-severe OSA. Sleep.

[31] Budhiraja R, Kushida CA, Nichols DA, Walsh JK, Simon RD, Gottlieb DJ, Quan SF (2017). Predictors of sleepiness in obstructive sleep apnoea at baseline and after 6 months of continuous positive airway pressure therapy. Eur Respir J.

[32] Malhotra A, Crocker ME, Willes L, Kelly C, Lynch S, Benjafield AV (2018). Patient engagement using new technology to improve adherence to positive airway pressure therapy: a retrospective analysis. Chest.

[33] Bouloukaki I, Giannadaki K, Mermigkis C, Tzanakis N, Mauroudi E, Moniaki V (2014). Intensive versus standard follow-up to improve continuous positive airway pressure compliance. Eur Respir J.

[34] Munafo D, Hevener W, Crocker M, Willes L, Sridasome S, Muhsin M (2016). A telehealth program for CPAP adherence reduces labor and yields similar adherence and efficacy when compared to standard of care. Sleep Breath.

[35] Li Z, Cai S, Wang J, Chen R (2022). Predictors of the efficacy for daytime sleepiness in patients with obstructive sleep apnea with continual positive airway pressure therapy: a meta-analysis of randomized controlled trials. Front Neurol.

[36] Thaler RH, Sunstein CR (2008). Nudge: improving decisions about health, wealth, and happiness.

[37] Lebret M, Arnol N, Martinot JB, Lambert L, Tamisier R, Pepin JL, Borel JC (2018). Determinants of unintentional leaks during CPAP treatment in OSA. Chest.

[38] Berry RB, Beck E, Jasko JG (2020). Effect of cloud-based sleep coaches on positive airway pressure adherence. J Clin Sleep Med.

[39] Chang J, Kim J, Becker K, Benjafield A, Crocker M, Woodrum R (2017). Impact of automated web-education and CPAP tele-monitoring on CPAP adherence at 3 months and 1 year. The tele-OSA randomized clinical trial. Sleep.

[40] Crocker M, Chang J, Liang J, Becker K, Kim J, Woodrum R (2017). Automated tele-monitoring on CPAP adherence at 1 year. The Tele-OSA trial. Eur Respir J.

[41] Lewis KE, Seale L, Bartle IE, Watkins AJ, Ebden P (2004). Early predictors of CPAP use for the treatment of obstructive sleep apnea. Sleep.

